# An updated h-index measures both the primary and total scientific output of a researcher

**DOI:** 10.15190/d.2015.42

**Published:** 2015-09-30

**Authors:** Octavian Bucur, Alexandru Almasan, Roman A. Zubarev, Mark T. Friedman, Garth L. Nicolson, Pavel Sumazin, Mircea Leabu, Barbara S. Nikolajczyk, Dorina Avram, Tanja Kunej, George A. Calin, Andrew K. Godwin, Hans-Olov Adami, Peter G. Zaphiropoulos, Des R. Richardson, Gerold Schmitt-Ulms, Håkan Westerblad, Megan Keniry, Georges E. R. Grau, Salvatore Carbonetto, Radu V. Stan, Aurel Popa-Wagner, Kasumov Takhar, Beverly W. Baron, Paul J. Galardy, Feng Yang, Dipak Datta, Oluwole Fadare, KT Jerry Yeo, Georgiana Roxana Gabreanu, Stefan Andrei, Georgiana R. Soare, Mark A. Nelson, Elisa A. Liehn

**Affiliations:** Department of Pathology, Harvard Medical School and Beth Israel Deaconess Medical Center, Boston, MA, USA; Department of Cancer Biology, Lerner Research Institute & Department of Radiation Oncology, Taussig Cancer Institute, Cleveland Clinic, Cleveland, Ohio, United States of America; Department of Medical Biochemistry and Biophysics, Karolinska Institutet, Stockholm, Sweden; Department of Pathology, St. Luke's-Roosevelt Hospital Center, Beth Israel Medical Center and Icahn School of Medicine at Mount Sinai, New York, NY, USA; Department of Molecular Pathology, The Institute for Molecular Medicine, Huntington Beach, CA, USA; Department of Pediatrics, Baylor College of Medicine, Houston, TX, USA; Department of Cellular and Molecular Medicine, University of Medicine and Pharmacy "Carol Davila" and "Victor Babes" National Institute of Pathology, Bucharest, Romania; Boston University School of Medicine, Department of Microbiology, Boston, MA, USA; Center for Cell Biology and Cancer Research & Center for Immunology and Microbial Disease, Albany Medical College, Albany, NY, USA; Department of Animal Science, Biotechnical Faculty, University of Ljubljana, Groblje 3, 1230, Domzale, Slovenia; Department of Experimental Therapeutics and Leukemia & Center for RNA Interference and Non-Coding RNAs, University of Texas MD Anderson Cancer Center, Houston, TX, USA; Department of Pathology and Laboratory Medicine, University of Kansas Cancer Center Kansas City, KS, USA; Department of Medical Epidemiology and Biostatistics, Karolinska Institute, Stockholm, Sweden; Department of Biosciences and Nutrition, Karolinska Institute, Sweden; Department of Pathology and Bosch Institute, University of Sydney, Sydney, Australia; Department of Laboratory Medicine & Pathobiology, University of Toronto, Medical Sciences Building, 6th Floor, 1 King's College Circle, Toronto, Ontario M5S 1A8, Canada; Tanz Centre for Research in Neurodegenerative Diseases, University of Toronto, Krembil Discovery Centre, 6th Floor, 60 Leonard Avenue, Toronto, Ontario M5T 2S8, Canada; Department of Physiology and Pharmacology, Karolinska Institute, Stockholm, Sweden; Department of Biology, University of Texas- Pan American, 1201 W. University Dr., Edinburg, TX 78539, USA; Department of Pathology, University of Sydney, Sydney, Australia; Centre for Research in Neuroscience, McGill University Health Sciences Centre, Montreal, Canada; The Geisel School of Medicine at Dartmouth, Dartmouth College, Lebanon, NH, USA; Department of Psychiatry, Rostock University Medical School, Rostock, Germany; Department of Gastroenterology and Hepatology, Cleveland Clinic, Cleveland, OH, USA; Department of Pathology, University of Chicago, Chicago, IL, USA; Department of Biochemistry and Molecular Biology & Division of Pediatric Hematology/Oncology, Mayo Clinic, Rochester, MN, USA; Department of Molecular and Cellular Biology, Baylor College of Medicine, Houston, TX, 77030, USA; CSIR-Central Drug Research Institute, Biochemistry Division, Lucknow, UP, India; Department of Pathology, University of California, San Diego, San Diego, CA, USA; Carol Davila University of Medicine and Pharmacy, Bucharest, Romania; CNHO des Quinze-Vingts, Service de Médecine Interne, Paris, France; Hopital Femme Mere Enfant, Hospice Civile de Lyon, Lyon, France; Department of Pathology, The University of Arizona, Tucson, AZ, USA; Institute for Molecular Cardiovascular Research (IMCAR), RWTH Aachen University, Germany

**Keywords:** Updated h-index, Hirsch index, h(p, t), h(p), h(t), primary scientific output

## Abstract

The growing interest in scientometry stems from ethical concerns related to the proper evaluation of scientific contributions of an author working in a hard science. In the absence of a consensus, institutions may use arbitrary methods for evaluating scientists for employment and promotion. There are several indices in use that attempt to establish the most appropriate and suggestive position of any scientist in the field he/she works in. A scientist’s Hirsch-index (h-index) quantifies their total effective published output, but h-index summarizes the total value of their published work without regard to their contribution to each publication. Consequently, articles where the author was a primary contributor carry the same weight as articles where the author played a minor role. Thus, we propose an updated h-index named Hirsch(p,t)-index that informs about both total scientific output and output where the author played a primary role. Our measure, h(p,t) = h(p),h(t), is composed of the h-index h(t) and the h-index calculated for articles where the author was a key contributor; i.e. first/shared first or senior or corresponding author. Thus, a h(p,t) = 5,10 would mean that the author has 5 articles as first, shared first, senior or corresponding author with at least 5 citations each, and 10 total articles with at least 10 citations each. This index can be applied in biomedical disciplines and in all areas where the first and last position on an article are the most important. Although other indexes, such as r- and w-indexes, were proposed for measuring the authors’ output based on the position of researchers within the published articles, our simpler strategy uses the already established algorithms for h-index calculation and may be more practical to implement.

## Introduction

The current scientific community needs strategies to rank the individual contribution of authors in order to most accurately appreciate the value of their efforts. Scientometry has developed as a useful field for objectively evaluating published scientific work, mainly in hard sciences, including biomedical research. However, as it is expected in science, a number of criticisms were proposed for many of the scientometric indices. Although none of these indices is perfect by itself, a complex assessment can be helpful to most accurately appreciate any author’s published work. Moreover, a careful evaluation is critical for the career of scientists, as well as for employers in academia and science alike.

## Why do we need an updated h-index?

The h-index, introduced by professor J.E. Hirsch in 2005, is now widely accepted and employed. The h-index of a scientist has index x if x of his/her published articles have at least x citations each and his/her remaining articles have ≤ x citations each^1^. The h-index is a measure of the total effective research output of a scientist^1^. However, the h-index does not take into consideration the order of the authors within the published articles, which in some cases can lead to over- or under-evaluation of the importance of an author’s scientific output^2,3^. An author’s rank is important in most research fields, including biomedical research, with the first and last (senior or corresponding) authors being the most important contributors to an article. Thus, an updated h-index that includes both a measure of the primary output of an author (as the first, shared first, senior or corresponding author) and the total output of the author (all articles regardless of an author’s position – the current h-index) is needed and would be a useful tool in the evaluation of biomedical publication output.

## Currently proposed indexes for evaluation of research output based on author rank

Other indexes have been proposed for evaluation of research output based on an author’s rank, including the r-index and w-index^2-4^. The *revised h-index (r-index)* for biomedical research was proposed in 2012 by professor A.A. Romanovski, where the first and last author positions are evaluated as being 4 times more important than middle author positions, within a published article^2^. Another index, the *weighted h-index (w-index)* was proposed in 2009 by professor C.T. Zhang^3^and is calculated taking into account the position of each author and giving a weighted coefficient of 1 for the first and last (corresponding) authors and linear decreasing coefficient numbers for the authors in positions 2, 3,…, n-1, where n is the number of the authors. Both methods are useful in many cases, but may prove inaccurate in some specific situations^2,3^, including ones where shared first authors or multiple corresponding/senior authors are concerned.

## Our proposed h(p,t) index measures both the primary output [h(p) index] and total output [h(t) index]

We propose here an updated Hirsch factor, termed Hirsch(p,t), or h(p,t), that calculates both the primary research output (h(p) index) and the total research output (h(t) index, currently known as h-index) of an author. h(p,t) = h(p),h(t) is composed of the h-index h(t) calculated for all articles published by the author, and the h-index h(p) calculated for articles where the author is a key contributor: i.e. first/shared first or last/shared last (senior or corresponding) author.

For example, an author with 10 published articles, of which 2 are as first author and on 1 he/she is the last (corresponding) author, each of the articles being cited more than 10 times, would have an h(p,t)-index of h(p,t)=2+1,10=3,10. This means that the author has 3 articles as first and last author with at least 3 citations each (primary research output), and 10 total articles with at least 10 citations each (total research output).

The h(p,t) index will be very helpful in distinguishing the authors with high primary research output from those with low primary research output, both when their total h indexes have similar or very distinct values.

## How will this updated h-index help the research community and evaluation of a researcher’s scientific output?

Despite its flaws, the currently used h-index is a useful way of measuring a researcher’s total publication output, whereas the primary output as first, shared first, last and shared last author is not measured^2,3,5,6^. Since the employed algorithm used for the h-index calculation is already recognized as useful by the scientific community and is widely used, we believe that addition of the primary research output information to this would be also useful. This can be done by using the same h-index calculation for the total output, adding primary output, and showing them together in the same term.

## Why do we think that the h(p,t) index should be employed by the research community?

Using an already accepted algorithm plus additional information on primary research output could allow the scientific community to accept this small, but significant improvement faster than would likely be the case with other proposed and less tested indexes. Moreover, other proposed indexes may miss the already proven usefulness of the h-index.

In our opinion, assigning weight coefficients for the position of authors within an article (r-index or w-index)^2,3^, although useful in many cases, is definitely prone to errors. For example, it is hard to correctly estimate and assign quantitative weight coefficients to compare the work of authors in the same manuscript, even when the contribution of each author is described (which is only true in a small number of journals). Assigning the same weight coefficient for the authors on the same position from different papers could be helpful, but it is not completely accurate. However, such quantitative indexes may complement h- or h(p,t)-indexes in the future.

It is worth pointing out that our strategy takes into consideration not only the first and last authors, but also the shared first or shared last (senior or corresponding) author positions. Although the automatic detection of the first and last author is not a problem, automatic detection of the shared first author or shared senior/corresponding authors by established databases, such as Scientific Citation Index, Google Scholar or Scopus, may be a challenge. A potential solution would be the enabling of limited editing by authors to manually identify shared authorship contributions to improve the accuracy of the h(p,t) index.

*In conclusion, we describe a useful and simpler strategy of evaluating researchers’ scientific output based on the calculation of the h-index for both the primary and total research output. This strategy can be applied in all fields of research where the first and last authors are the most important authors within published articles, including biomedical research.*

## KEY POINTS

**◊** ***Hirsch(p,t) = h(p,t) = h(p), h(t)***

**◊** ***h(p)*** *is the h-index calculated based on the articles as first/shared first and last/shared last author (**primary output**)*

**◊** ***h(t)*** *is the h-index calculated based on all articles published (known as h-index, or* ***total*** ***research output*** *)*

**◊** ***Example:*** ***h(p,t)=5,10*** *means that the author has an **h-index of 5 for his/her articles published as first and last author**, while **having an h-index of 10 for all articles***

## References

[1] Hirsch J E (2005). An index to quantify an individual's scientific research output.. Proceedings of the National Academy of Sciences of the United States of America.

[2] Romanovsky Andrej A (2012). Revised h index for biomedical research.. Cell cycle (Georgetown, Tex.).

[3] Zhang Chun-Ting (2009). A proposal for calculating weighted citations based on author rank.. EMBO reports.

[4] Sekercioglu Cagan H (2008). Quantifying coauthor contributions.. Science (New York, N.Y.).

[5] Saleem Taimur (2011). The Hirsch index - a play on numbers or a true appraisal of academic output?. International archives of medicine.

[6] Carbon Claus-Christian (2011). The Carbon_h-factor: predicting individuals' research impact at early stages of their career.. PloS one.

